# Integrated Bioinformatics Analysis of the Clinical Value and Biological Function of ATAD2 in Hepatocellular Carcinoma

**DOI:** 10.1155/2020/8657468

**Published:** 2020-05-05

**Authors:** Xiangyu Meng, Lu Wang, Bo Zhu, Jun Zhang, Shuai Guo, Qiang Li, Tao Zhang, Zhichao Zheng, Gang Wu, Yan Zhao

**Affiliations:** ^1^Department of Gastric Surgery, Cancer Hospital of China Medical University/Liaoning Cancer Hospital and Institute, Shenyang, Liaoning, China; ^2^Department of Ultrasonography, The Fourth Affiliated Hospital of China Medical University, Shenyang, Liaoning, China; ^3^Department of Information Management, The Information Center, Cancer Hospital of China Medical University/Liaoning Cancer Hospital & Institute, Shenyang, Liaoning, China; ^4^Department of Pathology, Cancer Hospital of China Medical University/Liaoning Cancer Hospital and Institute, Shenyang, Liaoning, China; ^5^Hepatobiliary Surgery Department and Unit of Organ Transplantation, The First Hospital of China Medical University, Shenyang, Liaoning, China

## Abstract

ATPase family AAA domain-containing protein 2 (ATAD2), a chromatin regulator and an oncogenic transcription cofactor, is frequently overexpressed in many cancers, particularly in hepatocellular carcinoma (HCC). By integrating open-access online mRNA datasets and our institutional tissue data on HCC, the clinical role and functions of ATAD2 were analyzed by bioinformatic algorithms. We systematically examined ATAD2 expression in HCC based on a large sample population, integrating data from our institution and the GEO, Oncomine, and TCGA datasets. Aberrant ATAD2 expression related to pathways was identified by bioinformatic algorithms. The effects of ATAD2 downregulation on the cycle cell were also determined. A pooled analysis from 28 datasets indicated that ATAD2 overexpression was found in HCC (SMD = 8.88, 95% CI: 5.96–11.81, *P* < 0.001) and was correlated with poor survival. Subgroup analysis of Asian patients with a serum alpha-fetoprotein (AFP) concentration < 200 ng/ml in stage I + II showed that the ATAD2-high group had a more unfavorable overall survival (OS) rate than the ATAD2-low group. The receiver operating characteristic curve indicated that the efficiency of ATAD2 for HCC diagnosis was considerable (area under the curve = 0.89, 95% CI: 0.86–0.91). Functional analysis based on bioinformatic algorithms demonstrated that ATAD2 participates in cell division, mitotic nuclear division, DNA replication, repair, and cell cycle processes. ATAD2 knockout in HCC cells downregulated cyclin C and cyclin D1 protein levels and resulted in G1/S phase arrest in vitro. The kinesin family member C1 (KIFC1), shugoshin 1 (SGO1), GINS complex subunit 1 (GINS1), and TPX2 microtubule nucleation factor (TPX2) genes were closely related to ATAD2 upregulation. ATAD2 may interact with TTK protein kinase (TTK) to accelerate HCC carcinogenesis. ATAD2 plays a vital role in HCC carcinogenesis by disturbing the interaction between chromatin proteins and DNA. Targeting ATAD2 represents a promising method for the development of therapeutic treatments for cancer.

## 1. Introduction

Hepatocellular carcinoma (HCC), constituting 90% of all primary liver tumors, is the fifth most malignant tumor worldwide. A total of 841,000 newly diagnosed cases and over 782,000 related deaths have been reported [1]. Due to its epidemiological features, HCC has received a lot of research attention. Although the surgical techniques, diagnostic methods, and combined treatments have greatly improved, patients diagnosed with HCC have a poor long-term prognosis, largely due to the high rates of intrahepatic metastasis (44.0–62.2%) and a 5-year survival rate of just 3% after surgical removal [2–4]. HCC is often diagnosed at advanced stages after the appearance of symptoms. A better understanding of the molecular mechanisms of hepatocarcinogenesis will therefore contribute to the development of a molecular target therapy for this type of cancer. Novel molecular biomarkers that can precisely evaluate disease progression and clinical results in the early stages of disease are urgently needed to facilitate an early diagnosis and for the development of personalized treatment.

ATAD2, an evolutionarily conserved AAA protein mapped to chromosome 8q24, possesses two AAA+ domains and a bromodomain (BRD) [5]. The unique structure of ATAD2 suggests that it plays an important role in regulating ATPase activity, protein multimerization, and binding to acetylated histones or nonhistones [6]. ATAD2 is often overexpressed in many human tumors, and its aberrant expression has been correlated with high histologic grades, poor overall survival (OS), tumor metastasis, and recurrence [7–10]. ATAD2 has also been identified as a coactivator of hormone-induced nuclear receptors (oestrogen receptor alpha (ER*α*) and androgen receptor (AR)), E2Fs, and c-myc for the promotion of tumor progression [5, 11, 12]. Previous studies have suggested that silencing ATAD2 may inhibit malignant tumor biological phenotypes, such as invasion, metastasis, and proliferation, consistent with our previous results regarding HCC [13]. These findings clearly indicate that the status of ATAD2 expression plays a vital role in the occurrence and prognosis of tumors, particularly HCC. Nonetheless, a comprehensive bioinformatics analysis of the functions of ATAD2 has not yet been performed. Thus, in the present study, we performed an integrated analysis of our institutional data and datasets from The Cancer Genome Atlas (TCGA), Gene Expression Omnibus (GEO), and Oncomine to determine the diagnostic value and functions of ATAD2 in HCC.

## 2. Materials and Methods

### 2.1. Patients Tissue Specimens

Tumor specimens (*n* = 80) and adjacent noncancerous tissues (*n* = 20) were obtained from patients with HCC who had only undergone surgical resection with curative intent at the First Affiliated Hospital of China Medical University from July 2012 to December 2014 (China Medical University date, CMUD). The pathological diagnoses and differentiations were confirmed by three independent pathologists according to the current classification system for HCC (World Health Organization). The clinicopathological features of all patients are provided in Table 1. Fresh specimens were snap-frozen in liquid nitrogen or stored at −80°C immediately after resection. This study was approved by the Institutional Ethics Committee of China Medical University. Written informed consent was obtained from all patients.

### 2.2. Immunohistochemistry and Assessment of Immunostaining

Immunohistochemistry was conducted as previously described [13]. ATAD2 expression in protein levels was scored semiquantitatively also according to the previous contents described [13, 14]. Briefly, samples were considered positive if the nucleus or cytoplasm of the sample cells presented a positive staining. The percent positivity and staining intensity were, respectively, defined as different scores. The scores of the percent positivity and the staining intensity were combined and assessed. Finally, the immunohistochemical ATAD2 staining was grouped into two categories: low expression and high expression.

### 2.3. TCGA Dataset

The mRNA expression datasets of the genes from the Cancer Genome Atlas datasets liver HCC (TCGA LIHC), containing 374 HCC samples and 50 normal samples, were downloaded for further analysis (http://cancergenome.nih.gov/). The clinical datasets from 374 patients with HCC, including age, gender, race, cirrhosis, histological type, family cancer history, grade, vascular tumor cell type, TNM stage, and pathological T/N/M stage, were also estimated. The data were used to assess the correlation between the ATAD2 mRNA expression levels and prognosis.

### 2.4. Oncomine and GEO Datasets

Other open online datasets, such as Oncomine (http://www.oncomine.org) and the Gene Expression Omnibus (GEO) (https://www.ncbi.nlm.nih.gov/geo/), were also checked for any HCC-relevant RNA-seq levels to analyze by combining the search terms “HCC”, “hepatocellular”, “liver”, “cancer”, “tumor”, and “malignant” using “or” or “and”. Further details on the microarray data and platforms are provided in Table 1.

### 2.5. Bioinformatics Analysis

The RNA-seq data downloaded from TCGA and GEO were analyzed using the edgeR package of R language (version 3.5.1) to identify the differentially expressed genes (DEGs) between HCC tissues and nontumor tissues. We defined *P* − adj < 0.05 and (∣log2*FC*∣) > 1 as the thresholds for screening the DEGs. The R package of “WGCNA” (Langfelder & Horvath, 2008) was used to construct a coexpression network for these DEGs in 374 HCC samples using the corresponding clinical information. We identified biologically significant modules using Pearson's correlation test to evaluate the association between modules and clinical features, including ATAD2 expression status. The module that showed the highest correlation with aberrant ATAD2 expression was selected. The Database for Annotation, Visualization and Integrated Discovery (DAVID) version 6.8 (https://david.ncifcrf.gov/) was used to categorize the gene data for the ATAD2-related target modules, namely, biological process, cellular component, and molecular function, using the FAT datasets of the Gene Ontology (GO) functional and pathway enrichment analysis using Kyoto Encyclopedia of Genes and Genomes (KEGG) and visualized using GraphPad Prism 7 (San Diego, CA, USA). Then, the gene significance (GS), high module membership (MM), and MCODE score for different genes in the gene modules of interest were calculated using the GS value, the *R* value from the MM correlation analysis, and the degree numbers from MCODE score analysis by ranking the top 30 genes. Based on the overlapping genes between the GS, MM, and MCODE scores, the hub genes were identified.

### 2.6. Liver Cancer Cell Line Cell Cultures and Construction of RNAi Lentivirus Vector

The liver cancer cell lines HepG2 and Bel-7402 were obtained from the Shanghai Cell Bank (Shanghai, China). HepG2 and Bel-7402 were grown in DMEM (Gibco, USA). All media were supplemented with 10% fetal bovine serum (FBS = (Invitrogen) and 100 U/ml penicillin (Sigma, St. Louis, MO). The RNAi lentivirus vector for the downregulation of ATAD2 expression was constructed and transfected into the HepG2 and Bel-7402 cells, as previously reported [14].

### 2.7. Cell Cycle Analysis

The HepG2 and Bel-7402 cells were placed in 6-well plates and transfected with ATAD2-RNAi-lentivirus or control. The cells were seeded at a density of 5 × 10^5^ per well and trypsinized. Cell cycle analysis was performed after staining with propidium iodide (Keygene, China). The cell cycle distribution was quantified using a flow cytometer.

### 2.8. Western Blotting

The cells were lysed with RIPA buffer (Beyotime, China) and centrifuged at 12,000 g for 30 min at 4°C. The protein concentration was determined using the Bradford method in a SpectraMax M5 multidetection reader (MDS, USA) at 562 nm. Equal amounts of protein (10 *μ*g) were run on 10% to 12% SDS-PAGE acrylamide gels and transferred to PVDF membranes (Millipore, Billerica, MA, USA). After the blocking of nonspecific binding sites for 2 h with 5% nonfat milk in TBST, the membrane was incubated overnight at 4°C with primary antibodies, including anti-ATAD2 (Abcam, UK), anticyclin D1, anticyclin C (Proteintech, USA) (both diluted at 1 : 1,000). The membranes were then incubated with secondary antibodies for 1 h at room temperature. GAPDH was used as the loading control for the human samples. The immunoblots were visualized using an ECL system (Millipore, Bedford, MA, USA).

### 2.9. Statistical Analysis

SPSS 23.0 (SPSS Inc., Chicago, IL, USA) software for Windows was used for the statistical analysis. The association between ATAD2 expression and the clinicopathological features of the HCC patients were evaluated using the Chi-squared (*χ*^2^) test. The data for ATAD2 expression were presented as the mean ± standard deviation (SD) in our Center for each of the datasets. The independent-samples *T* test was used to compare the differential ATAD2 expression levels in the different patients (HCC vs. normal) and the clinicopathological features. In order to assess the overall diagnostic value of ATAD2 from 28 databases to distinguish the HCC patients from the controls, the pooled sensitivity, specificity, diagnostic odds ratio (DOR), the summary receiver operator characteristic (SROC) curve, and the area under the curve (AUC) were calculated using STATA 12.0. The sensitivity and specificity of each biomarker (ATAD2, GPC3, and AFP) for the diagnosis of HCC and the AUC value with 95% CI were calculated using GraphPad Prism 5 (San Diego, CA, USA). The coincidence rate was determined as follows: (true positive + true negative)/(true positive + true negative + false positive + false negative). The differential expression levels of ATAD2, the ROC curves, and the cell cycle results were also visualized using GraphPad Prism 5. The Kaplan-Meier method was used to calculate the patients' survival using the log-rank test. A Cox repression model was performed for the univariate and multivariate analysis of the prognostic variables. All *P* values were two-sided, and *P* < 0.05 was considered statistically significant. A comprehensive perspective on ATAD2 expression was analyzed by integrating open online data in the form of meta-analysis using STATA 12.0 (StataCorp, College Station, TX, USA). The total SMD (Standard Mean Difference) was computed. When SMD > 0 and its 95% CI did not cross, an integer of 0 indicated that ATAD2 was significantly overexpressed in tumors compared to nontumor tissues. The comprehensive efficiency of ATAD2 in distinguishing tumor from nontumor tissues was determined using SROC curves. The overall design of the present study is shown in Figure S1 in the form of a flow chart.

## 3. Results

### 3.1. ATAD2 Expression and Its Relationships with Clinicopathological Features in HCC Based on the CMUD and TCGA LIHC Datasets

**Figure 1:**
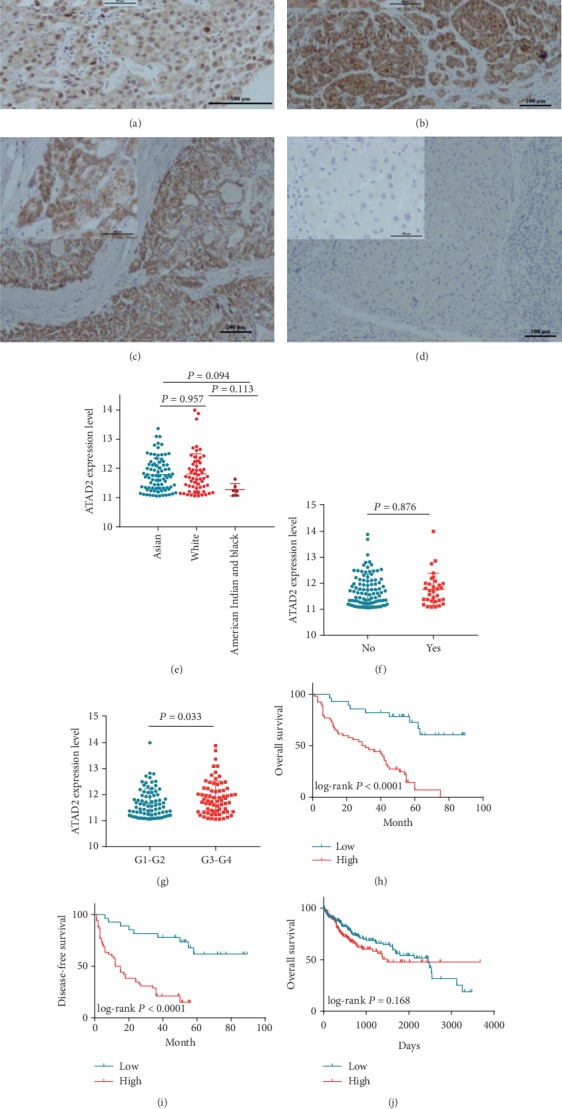
IHC staining of ATAD2 in HCC tissues and expression and prognosis of ATAD2 in HCC based on TCGA and our institutional data. Positive immunohistochemical reaction was showed as brown staining. Representative tissue sections with different immunochemical staining status (from nucleus to negative) of ATAD2: (a) nucleus: poorly differentiated; (b) cytoplasm: moderately poorly differentiated; (c) less cytoplasm: moderately differentiated; (d) negative: well differentiated. For each intensity group, the specimens were obtained (×200 and ×400 magnification). (e) Scatter plot of ATAD2 expression at different races. (f) Scatter plot of ATAD2 expression based on family cancer history. (g) Scatter plot of ATAD2 expression at different histological grades. (h) Overall survival of HCC patients related to ATAD2 status (protein level) in institutional data. (i) Disease-free survival of HCC patients related to ATAD2 status (protein level) in institutional data. (j) Overall survival of HCC patients related to ATAD2 status (mRNA level) based on TCGA data.

**Figure 2:**
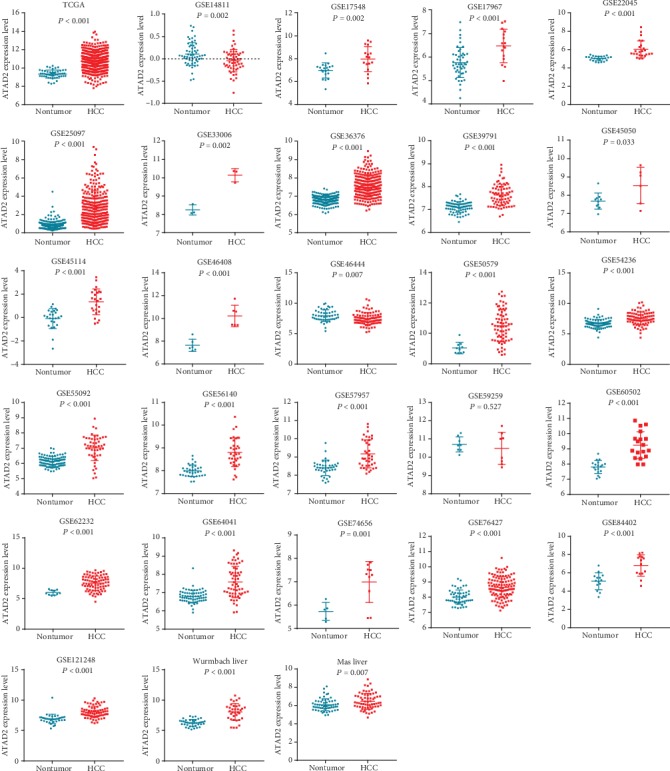
Levels of ATAD2 expression in HCC vs. nontumor tissues based on 28 datasets.

We first explored the expression of ATAD2 mRNA in 33 types of tumors based on gene expression profiling interactive analysis (GEPIA). As shown in Figure S2A, ATAD2 expression was increased in 17 HCC tumors. To further detect the expression of ATAD2 in HCC, the CMUD and TCGA LIHC datasets were analyzed. The CMUD results suggested that ATAD2 was overexpressed in a larger percentage (65%, 52/80) of HCC samples compared to nontumor liver specimens by *in situ* hybridization (ISH) (30%, 6/20, Figure S3). The high levels of ATAD2 protein were correlated with tumor size (*P* = 0.018), metastasis (*P* = 0.009), serum alpha-fetoprotein (AFP) (*P* = 0.010), and TNM stage (*P* = 0.033, Table 2). We also found that the specimens with metastasis and a high TNM stage accumulated ATAD2 protein in the cytoplasm and/or nucleus (Figures 1(a)–1(d)) by immunohistochemistry (IHC). In the TCGA data, which contained RNA-seq and clinical datasets from 374 HCC patients and 50 nontumor patients, ATAD2 mRNA expression was upregulated in the HCC samples compared to the nontumor samples (10.75 ± 0.05668 vs. 9.338 ± 0.06266; *P* < 0.0001, Figure 2). The overexpression of ATAD2 was correlated with race (*P* = 0.008), a family history of cancer (*P* = 0.018), and tumor grade (*P* < 0.001) (Table 3). Patients with G3-G4 had higher levels of ATAD2 expression than patients with G1-G2 (11.88 ± 0.07667 vs. 11.67 ± 0.06207; *P* = 0.033, Figure 1(g)). However, we did not observed any differential expression of ATAD2 mRNA among different races or family cancer histories (Figures 1(e) and 1(f)). In addition, no associations between sex, age, cirrhosis, histological type, tumor status, vascular tumor cell type, TNM stage, or T/N/M stage were observed (*P* > 0.05, Tables 2 and 3).

### 3.2. ATAD2 Expression Is Associated with the Prognosis of HCC

Furthermore, the prognostic value of aberrant ATAD2 expression was examined in the CMUD and TCGA datasets. Univariate analysis indicated that ATAD2 expression status, tumor size, metastasis, serum AFP, and TNM stage unfavorably influenced OS (Table S1). OS and disease-free survival (DFS) were significantly lower in patients with ATAD2 overexpression than in those with low ATAD2 expression (*P* < 0.001, Figures 1(h) and 1(i)). In the tumor size and metastasis subgroup, patients with high ATAD2 levels had a poorer prognosis than those with low ATAD2 levels, regardless of tumor size or metastasis status (Figures S4A–S4D). Other subgroup analyses among patients with a serum AFP concentration < 200 ng/ml or stage I + II revealed that the ATAD2-high group had a more unfavorable OS than the ATAD2-low group (both *P* < 0.0001) (Figure S4). The multivariate analysis showed that the ATAD2 expression status, serum AFP concentration, and metastasis were significant prognostic factors for HCC patients (Table S1). Unfortunately, the OS curve from the ATAD2 mRNA high expression group was not different from that of the low expression group in the TCGA dataset (HR = 0.774, 95% CI: 0.531–1.127; *P* = 0.168, Figure 1(j)). However, the subgroup analysis among patients of the Asian race revealed that the ATAD2-high group was associated with an unfavorable OS (*P* = 0.020) (Figure S4).

### 3.3. ATAD2 mRNA Expression in the GEO and Oncomine Databases

**Figure 3:**
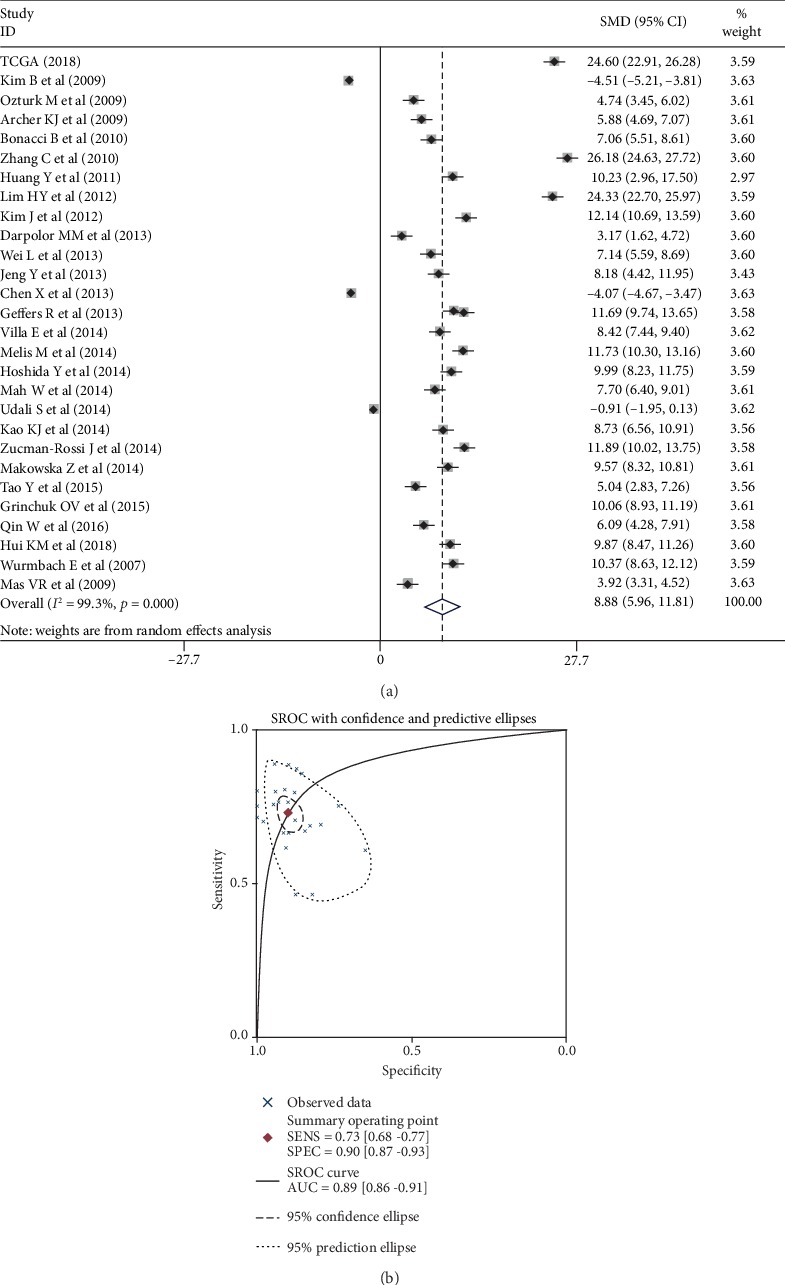
A meta-analysis of ATAD2 differential expression in HCC vs. nontumor tissues based on 28 datasets. (a) Forest plot on ATAD2 expression between HCC and nontumor tissues. (b) SROC curves for ATAD2 differential expression in HCC patients from nontumor tissues.

To detect ATAD2 mRNA expression in other HCC-related databases, 27 databases, including GEO and Oncomine, were searched, yielding a total of 1,569 HCC samples and 1,356 nontumor samples. All the ATAD2 mRNA expression values are provided in Table 3. The results suggested that ATAD2 mRNA expression was significantly increased in 1,417 HCC samples compared to that in 1,244 nontumor samples, except in the GSE14811, GSE46444, and GSE59259 datasets. No differences in ATAD2 expression between the HCC and nontumor groups in the GSE59259 dataset were observed (*P* = 0.593). Notably, the nontumor samples had higher levels of ATAD2 expression than the HCC samples in the GSE14811 and GSE46444 datasets (*P* = 0.002, *P* = 0.007). Scatter plots were drawn to visually represent the results (Figure 2). To explain the pooled analysis based on all data referring to ATAD2 expression in this article, a meta-analysis was conducted to comprehensively integrate the ATAD2 expression results from different databases. The pooled SMD reached 8.88 (95% CI: 5.96–11.81; *P* < 0.001) using the random effects model (Figure 3(a)), indicating that ATAD2 is significantly overexpressed in HCC, which was based on its high percentage of amplification (68/110, 61.8%) and mRNA upregulation (31/110, 28.2%) in genetic alterations from cBioPortal (Figure S2B).

### 3.4. Analysis of the Diagnostic Value of High ATAD2 Expression Based on TCGA, GEO, and Oncomine Databases

**Figure 4:**
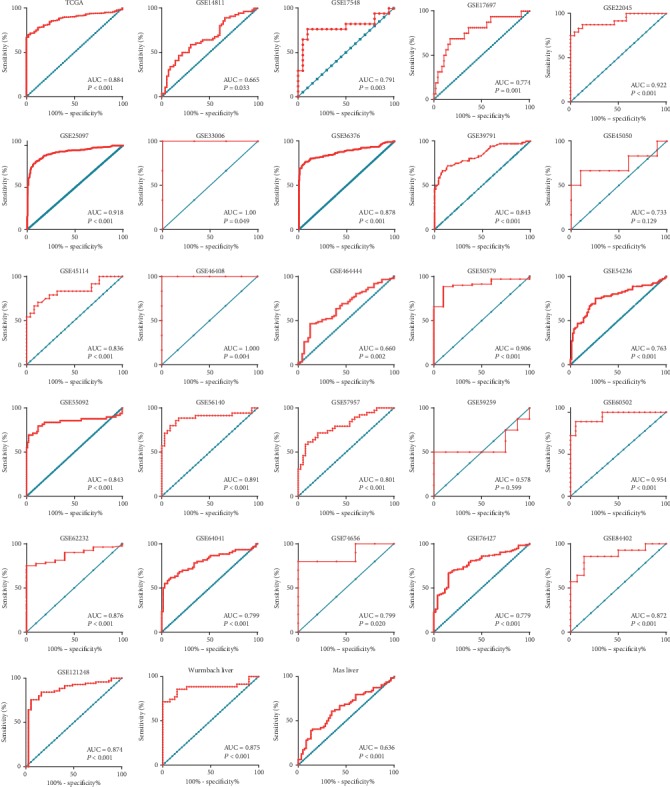
ROC curves of ATAD2 expression in HCC vs. nontumor tissues based on 28 datasets.

The diagnostic value of ATAD2 expression in HCC was assessed based on the AUC of the ROC curve. In the TCGA dataset, the AUC of the ROC was 0.8839, with a cut-off value of 10.15 and a sensitivity and specificity of ATAD2 in diagnosing HCC of 70.32% and 98%, respectively (Figure 3). Glypican-3 (GPC3) is an important member of the glypican family and has been previously used as a marker for the early diagnosis of HCC [15]. As such, we compared its diagnostic performance among ATAD2, GPC3, and AFP (a traditional biomarker for HCC diagnosis) in 165 Asian cases (159 tumors and 6 nontumors) on the TCGA database. The results suggested that the specificity of ATAD2, GPC3, and AFP was 100%. The sensitivity of ATAD2 and GPC3 was 83.02% and 84.91%, respectively, which were both higher than that of AFP. The difference was statistically significant. Similar results were also observed when comparing the coincidence rate and area under the ROC curve. However, there was no significant difference between ATAD2 and GPC3 in terms of sensitivity, coincidence rate, and area under the ROC curve (Table S2). In addition, the ROC curve plots of the GEO and Oncomine datasets were also drawn to visually represent the results (Figure 4). These results showed that ATAD2 overexpression had significant diagnostic value in HCC. Furthermore, the diagnostic accuracy was further evaluated by plotting the SROC and calculating the AUC, which was 0.89 (95% CI: 0.86–0.91). The pooled sensitivity, specificity, and DOR were 0.73 (95% CI: 0.68–0.77), 0.90 (95% CI: 0.87–0.93), and 8.88 (95% CI: 5.96–11.81), respectively. These results indicated that ATAD2 had a better discriminatory test performance and high diagnostic accuracy in distinguishing patients with HCC from healthy controls, similar to GPC3 (Figures 3(a) and 3(b)).

### 3.5. Identification of Aberrant ATAD2 Expression Related to Pathways by Bioinformatic Algorithms

**Figure 5:**
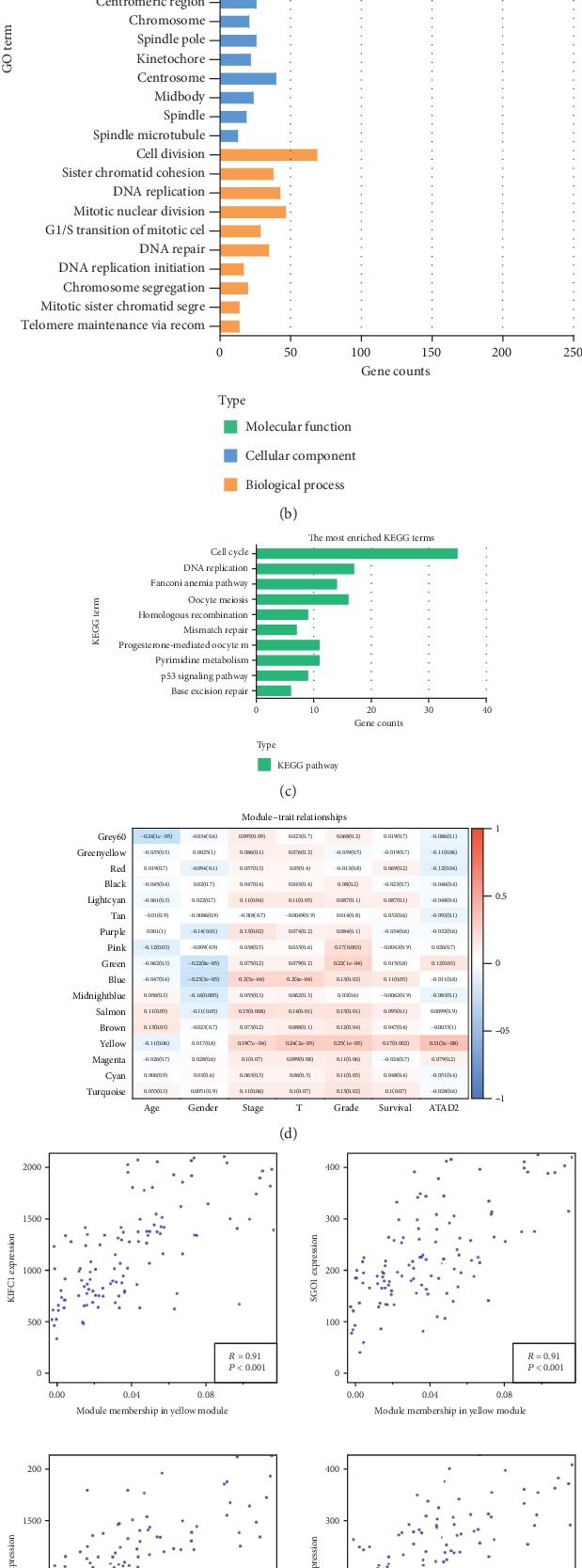
Identification of ATAD2-related genes and GO and KEGG pathway analysis of the ATAD2-related genes in HCC. (a) Volcano plot of the differential expression genes in HCC vs. normal liver tissues based on TCGA. Red indicates high expression and green indicates low expression (∣log2FC∣ > 1 and adjusted *P* value <0.05). (b, c) The *x*-axis showed the number of genes and the *y*-axis showed the GO and KEGG terms; (d) Heat map of the correlation between module eigengenes and clinical traits including ATAD2 aberrant expressions of HCC by using the WGCNA package of R language: each row indicated a module eigengene, and each column indicated a clinicopathological parameter. Each block contained the corresponding correlation coefficient and *P* value. (e) Scatter plot of module eigengenes related to ATAD2 aberrant expression in the yellow set. (f) Hub genes were filtered out with module membership (MM), gene significance (GS), and MCODE degree. (g) Protein-protein interaction network of ATAD2, TTK, KIFC1, SGO1, GINS1, and TPX2 in the yellow set.

To gain further insights into the biological functions associated with ATAD2 upregulation, WGCNA algorithms were used. As a gene screening method, WGCNA analysis can be used to simplify complex microarray data into several functional modules correlated with clinical traits, which are composed of coexpressed genes. Subsequently, hub genes in the most prominent modules related to clinical traits were filtered and analyzed. Firstly, the DEGs were screened by defining *P* < 0.05 and ∣logFC∣ ≥ 1 as the thresholds for 374 HCC samples in the TCGA database. In total, 9,219 DEGs were used to construct a coexpression network with the WGCNA package to identify a module of genes related to aberrant ATAD2 expression, which was calculated as a clinicopathological feature to be analyzed (Figure 5(a)). The module of genes shown in yellow, which includes 438 genes, was significantly related to ATAD2 expression (Figure 5(d)). To further investigate the functional associations of ATAD2-related genes, all 438 DEGs in the yellow module were submitted to the online database DAVID to identify representative GO terms and KEGG pathways [16] to elucidate the functional properties of the ATAD2-related DEGs. The top 10 GO and KEGG pathways sorted by the false discovery rate (FDR) are shown in Figures 5(b) and 5(c). The results showed that the yellow module of genes contained biological processes mainly enriched in cell division, mitotic nuclear division, DNA replication, and DNA repair. The cellular components were enriched mainly in the nucleus, nucleoplasm, and centrosome. The molecular functions were enriched mainly in protein binding, ATP binding, DNA binding, and chromatin binding (Figure 5(b)). These results suggest that ATAD2 acts as a “nuclear factor” to maintain the physiological function of chromatin and DNA. Likewise, KEGG pathway analysis also showed that ATAD2-related DEGs were enriched in the cell cycle, DNA replication, oocyte meiosis, Fanconi anemia pathway, homologous recombination, mismatch repair, pyrimidine metabolism, the p53 signalling pathway, and base excision repair, which are significantly related to the synthesis, assembly, and repair of genetic material (Figure 5(c)). Remarkably, the KIFC1, SGO1, GINS1, and TPX2 genes were mostly related to aberrant ATAD2 expression in the yellow module of genes, whose correlation analysis *R* values were all greater than 0.9 (Figure 5(e)). Furthermore, according to the GS, MM, and MCODE results, we sorted the ATAD2-related DEGs (yellow module) and selected 30 members of each group to derive the intersection (Figure 5(f)). Finally, TTK was identified as the hub gene. Notably, the protein-protein interaction (PPI) network indicated that ATAD2 may interact with TTK (Figure 5(g)).

### 3.6. Downregulation of ATAD2 Resulted in G1 Phase Arrest in HCC Cell Lines

**Figure 6:**
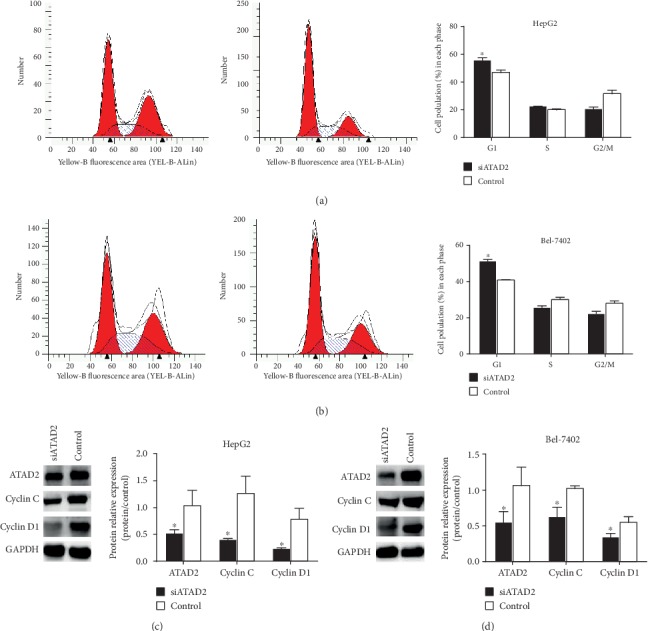
(a, b) RNAi lentivirus-mediated ATAD2 knockdown reduced HepG2 and Bel-7402 cell proliferation and led to a G1 phase cell cycle arrest. (c, d) cyclin C and cyclin D1 expressions in protein levels were detected when ATAD2 gene was downregulated by siRNA in HepG2 and Bel-7402 cell lines.

Based on the KEGG pathway analysis results, cell cycle tests were performed to further evaluate the effect of ATAD2 expression on cell cycle distribution. As shown in Figures 6(a) and 6(b), the percentage of cells in G1 phase increased, while the percentage of cells in S phase decreased in HepG2 and Bel-7402 cells when ATAD2 expression was downregulated. We also examined cyclin C and cyclin D1; both of which are required for the G1/S transition of the cell cycle at the protein level. Western blotting indicated that the expression of cyclin C and cyclin D1 was decreased after the knockdown of the ATAD2 gene in HepG2 and Bel-7402 cells (Figures 6(c) and 6(d)). These findings indicated that aberrant ATAD2 expression was correlated with the cell cycle process from the KEGG analysis, which was also consistent with the results from our previous study.

## 4. Discussion

HCC is the second leading cause of cancer-related death in East Asia, with over 50% of new cases being diagnosed in China. Chronic viral hepatitis and alcohol consumption are recognized as the two most important risk factors for the development of liver cirrhosis and, subsequently, HCC on a global scale. The development of HCC is complex and involves sustained inflammatory damage leading to hepatocyte necrosis, regeneration, and fibrotic deposition [17]. A failure to distinguish between groups at risk and the healthy population has been a leading factor in the delay of HCC diagnosis. Once diagnosed in the advanced stages, there are fewer curative treatment options available for patients. In addition to identifying HCC-related risk factors, the screening of potential candidates for clinical diagnosis and targeted therapy is necessary. As a conserved AAA ATPase and BRD factor, ATAD2 has received attention because of its vital functions in chromatin modification, protein complex assembly, and the promotion of gene transcription activation [5, 11, 18–21]. Despite these vital functions, ATAD2 has not been identified as a critical component in many molecular mechanism studies undertaken in recent years. Moreover, low levels of ATAD2 expression were reported after a comparison of metastatic and nonmetastatic breast cancer patients and controls, which differed from plausibly consistent viewpoints that ATAD2 was highly expressed in many cancers [22]. We also found that ATAD2 expression in different HCC datasets from the GEO did not remain stable. Hence, based on a large-scale sample size, which included data from our institution and from open-access online datasets, the expression of ATAD2 may be a reliable and valuable indicator. In addition, the positive research progress on small molecule inhibitors acting on BRDs has made it possible to target these BRD-containing proteins, such as ATAD2, for the diagnosis and treatment of HCC [23, 24].

In this study, we first systematically examined the expression of ATAD2 in HCC based on a large sample population, integrating data from our institution and from the GEO, Oncomine, and TCGA datasets. A pooled analysis showed that ATAD2 was significantly overexpressed in almost all HCC-related datasets. High ATAD2 expression was positively related to tumor size, metastasis, serum AFP concentration, and TNM stage in our study. The TCGA dataset results indicated that a high level of ATAD2 expression was closely correlated with race, a family cancer history, tumor grade, and disease stage. Both datasets highlighted the associations between aberrant ATAD2 expression and poor clinicopathological characteristics. Similar results have also been found in other ATAD2-related studies, such as those on colorectal cancer [25], gastric cancer [9], and cervical cancer [26], in which overexpressed ATAD2 levels often existed in more aggressive tumor subgroups. Likewise, the analysis of the TCGA dataset indicated that patients with G3-G4 had higher ATAD2 levels than those with G1-G2, supporting these findings. Furthermore, high levels of ATAD2 expression in HCC are a strong and independent predictor of shortened OS and DFS, although this result was not achieved when analyzing the TCGA dataset. These contradictory results could be explained by the subgroup analysis of ATAD2 expression on race from the TCGA dataset. However, we found that aberrant ATAD2 expression was closely related to different populations in the TCGA dataset, and a high level of ATAD2 expression among patients of the Asian race unfavorably influenced OS compared to a low level of ATAD2 expression. The results above were similar to those obtained from our institutional ISH sample, which consisted of Asian individuals. Subgroup analyses were also conducted. Tumor size and metastatic status seemed to have little effect on the prognostic value of ATAD2 expression status on OS. However, among patients with a serum AFP concentration <200 ng/ml or stage I + II, the ATAD2-high group had a worse prognosis than the ATAD2-low group (both *P* < 0.0001). These results correlate with those previously reported by Hwang [27].

In view of the aggressive biological features of ATAD2 emerging in tumors, particularly in HCC, we explored the possible clinical usage of ATAD2 for the detection and diagnosis of HCC. By combining the data on tissue ATAD2 levels from open datasets, including TCGA, GEO, and Oncomine, which included 1,569 HCC samples and 1,356 controls, we found that ATAD2 had a moderate diagnostic accuracy for HCC. As shown in Figure 5, the AUCs of the ROC curves in 23 of 28 datasets were greater than 0.75. A significant diagnostic value of ATAD2 expression in the tissues of patients with HCC compared to the healthy controls was observed. In addition, this diagnostic value of ATAD2 for HCC was also observed in comparison with GPC3 and AFP. Compared with AFP, a traditional detection marker, both ATAD2 and GPC3, has shown advantages over AFP in the diagnosis of HCC, even though this advantage seems to be more apparent in GPC3. However, when hepatitis occurs, the expression level of GPC3 in liver cells also increased [28]. To some extent, this increased the false positive rate of HCC diagnosis, resulting in a biased diagnosis. As a novel biomarker, the expression of ATAD2 in HCC was not associated with hepatitis cirrhosis in our own data, which indicated the advantages of ATAD2 over GPC3 for the diagnosis of HCC. More importantly, the SROC results also showed that ATAD2 yielded an AUC of 0.89, indicating that the efficiency of ATAD2 for HCC diagnosis was considerable, with upper-moderate sensitivity and specificity. As in AFP and GPC3 detection, ATAD2 may become a prospective molecular biomarker, allowing for the early diagnosis and treatment of HCC patients, thereby avoiding the adverse effects of tumor progression.

Normally, ATAD2 gene expression is tightly regulated by mitogenic signalling in proliferating cells, a process that is prominently observed in male germ cells and embryonic stem (ES) cells [12, 29]. Dysregulated control may cause ATAD2 upregulation, which can ultimately lead to carcinogenesis [12, 30]. A loss of inhibition upstream or by specific genetic modifications plays a vital role in aberrant ATAD2 expression. For instance, our previous study demonstrated that as an antioncogene, miR-372 was an upstream target of ATAD2, which was bound directly to its 3′ untranslated region (3′ UTR). In HCC, low miR-372 expression caused by the methylation of CpG in the promoter region ultimately led to the upregulation of ATAD2 expression [13, 31]. In addition to the posttranscriptional level of regulation, PPIs also play a part in ATAD2 expression. Both E2F1 and AR may enhance ATAD2 expression by acting at the ATAD2 promoter [11, 32]. Moreover, ATAD2 also acts as a direct target of certain protooncogenes, such as ACTR, AIB1, and SRC-3, integrating and/or activating multiple oncogenic programs or pathways to enhance tumorigenesis [7, 19, 33]. Of course, the amplification of the ATAD2 gene locus is also essential [34], as our cBioPortal analysis revealed that ATAD2 had a high percentage of amplification (68/110, 61.8%) among the genetic alterations. Regardless of the pathways that cause dysregulation, ATAD2 overexpression indeed damaged the maintenance of the ATAD2-related physiological process. Previous studies have suggested that ATAD2 functions as a transcriptional regulator and participates in the management of gene expression. As a coactivator of hormone receptors (ER*α* and AR) and c-myc, ATAD2 directly mediates the transcription of hormone receptor-related and c-myc pathway target genes [1, 3, 12]. The results of the GO analysis of ATAD2 molecular functions indicate the structural basis of the transcriptional function of ATAD2.

In addition to managing transcription, ATAD2 also appears to be involved in very specific and unique cellular processes, such as DNA repair and chromatin remodelling. By using a bioinformatics-based analysis, we observed that ATAD2 overexpression was closely related to cell division, mitotic nuclear division, DNA replication, and DNA repair. According to the ISH results of the nuclear-expressed protein, the cellular components identified by the GO analysis were enriched mainly in the nucleus, nucleoplasm, and centrosome. Cell cycle tests in the HepG2 and Bel-7402 HCC cells also demonstrated that ATAD2 and its related DEGs participate in cell cycle processes. These results were also consistent with Ciro's and Mjelle's findings, in which ATAD2 was consistently highly expressed in G1/S phase [5, 35]. Importantly, cyclin C and cyclin D1 protein expression was significantly inhibited when ATAD2 was not expressed. This evidence indicates that ATAD2 plays a vital role in regulating the cell cycle. In addition, based on the unique structure of ATAD2 itself, we also believe that ATAD2 acts as a generalist facilitator via protein recruitment at acetylated chromatin regions or via multimerization by its ATPase functions. Furthermore, the analysis of the KIFC1, SGO1, GINS1, and TPX2 genes revealed that these genes are mostly related to aberrant ATAD2 expression, which may explain the interactions between them, although further experiments are needed. By integrating multiple datasets, TTK, a dual-specificity protein kinase, was identified and may interact with ATAD2 based on the PPI network analysis. TTK is a component of the spindle assembly checkpoint, a surveillance mechanism that ensures the fidelity of chromosome segregation [36]. Previous studies have shown that TTK is often overexpressed in many cancers and that the upregulation of TTK is correlated with poor survival [37, 38]. The inhibition of TTK kinase activity has been previously found to lead to chromosome segregation errors, resulting in cancer [39, 40]. King et al. [41] found that high TTK expression levels accelerated the TGF-*β* signalling pathways to induce the epithelial-to-mesenchymal transition (EMT) in triple-negative breast cancer. The relationships between ATAD2 and TTK should be further tested. Thus, it was observed that ATAD2 is involved in controlling the key components of DNA repair, cell proliferation, and metastasis pathways, eventually resulting in more aggressive tumors. However, with the continuous progress of drug research, some small molecule inhibitors of ATAD2's BRD have been identified as novel drugs.

Although quite dissimilar to other druggable BRDs, ATAD2 has been considered “difficult to drug” because of its unique BRD structure [42]. Thus, it appears that more work is needed before the ATAD2 protein can become a promising target for the treatment of cancers in the future.

## 5. Conclusions

In the present study, we used integrated bioinformatics datasets with data from our institution to find that ATAD2 was overexpressed in HCC. High ATAD2 expression levels were positively related to aggressive phenotypes and poor survival in HCC. ATAD2 showed significant diagnostic value in patients with HCC. Functional GO and KEGG analyses suggested that ATAD2 overexpression participates in cell cycle regulation. ATAD2 might interact with TTK to accelerate HCC carcinogenesis.

## Figures and Tables

**Table 1:** Characteristics of datasets included in this study.

First author (publication year)	Country/region	Dataset	Platform	Samples	Cancer			Nontumor		
					N	Mean	SD	N	Mean	SD
Kim B et al. (2009)	South Korea	GEO:GSE14811	KRIBB GLP8177	Pair-matched HCC vs. adjacent liver tissues	56	-0.040	0.033	56	0.111	0.034
Ozturk M et al. (2009)	Turkey	GEO:GSE17548	Affymetrix GPL570	Cirrhosis liver vs HCC	17	7.967	0.267	20	6.968	0.148
Archer KJ et al. (2009)	USA	GEO:GSE17967	Affymetrix GPL571	Cirrhosis liver with or without HCC	16	6.477	0.179	47	5.768	0.094
Bonacci B et al. (2010)	USA	GEO:GSE22045	Affymetrix GPL570	Pair-matched HCC vs. adjacent liver tissues	24	6.034	0.191	24	5.047	0.051
Zhang C et al. (2010)	USA	GEO:GSE25097	Affymetrix GPL10687	Cirrhotic and normal livers vs HCC	268	2.818	0.109	289	0.788	0.024
Huang Y et al. (2011)	Taiwan	GEO:GSE33006	Affymetrix GPL570	Pair-matched HCC vs. adjacent liver tissues	3	10.150	0.209	3	8.246	0.160
Lim HY et al. (2012)	South Korea	GEO:GSE36376	Illumina GPL10558	Pair-matched HCC vs. adjacent liver tissues	240	7.576	0.040	193	6.806	0.016
Kim J et al. (2012)	USA	GEO:GSE39791	Illumina GPL10558	Pair-matched HCC vs. adjacent liver tissues	72	7.636	0.055	72	7.106	0.028
Darpolor MM et al. (2013)	USA	GEO:GSE45050	Affymetrix GPL6244	Cirrhotic and normal livers vs HCC	6	8.532	0.406	10	7.683	0.141
Wei L et al. (2013)	China	GEO:GSE45114	CapitalBio GPL5918	Normal vs. HCC and pericancer tissues	24	1.341	0.226	25	-0.086	0.171
Jeng Y et al. (2013)	Taiwan	GEO:GSE46408	Agilent GPL4133	Pair-matched HCC vs. adjacent liver tissues	6	10.210	0.386	6	7.636	0.221
Chen X et al. (2013)	USA	GEO:GSE46444	Illumina GPL13369	Liver tissues vs. HCC from HCC patients	88	7.478	0.106	48	7.961	0.139
Geffers R et al. (2013)	Germany	GEO:GSE50579	Agilent GPL14550	Liver cell and normal liver vs. HCC	70	10.500	0.126	10	9.037	0.118
Villa E et al. (2014)	Italy	GEO:GSE54236	Agilent GPL6480	Pair-matched HCC vs. adjacent liver tissues	81	7.534	0.119	80	6.689	0.077
Melis M et al. (2014)	USA	GEO:GSE55092	Affymetrix GPL570	Cirrhosis liver vs HCC	49	7.016	0.119	91	6.132	0.034
Hoshida Y et al. (2014)	USA	GEO:GSE56140	Illumina GPL18461	Cirrhosis liver vs HCC	35	8.806	0.106	34	7.991	0.044
Mah W et al. (2014)	Singapore	GEO:GSE57957	Illumina GPL10558	Pair-matched HCC vs. adjacent liver tissues	39	9.173	0.123	39	8.410	0.067
Udali S et al. (2014)	Italy	GEO:GSE59259	NimbleGen GPL18451	Alcohol-cirrhosis liver vs HCC	8	10.490	0.310	8	10.710	0.145
Kao KJ et al. (2014)	Taiwan	GEO:GSE60502	Affymetrix GPL96	Pair-matched HCC vs. adjacent liver tissues	18	9.241	0.209	18	7.808	0.101
Zucman-Rossi J et al. (2014)	France	GEO:GSE62232	Affymetrix GPL570	Normal vs. HCC	81	7.569	0.131	10	6.013	0.130
Makowska Z et al. (2014)	Switzerland	GEO:GSE64041	Affymetrix GPL6244	Normal vs. HCC	60	7.589	0.111	65	6.796	0.043
Tao Y et al. (2015)	China	GEO:GSE74656	Affymetrix GPL16043	Pair-matched HCC vs. adjacent liver tissues	10	6.990	0.278	5	5.727	0.173
Grinchuk OV et al. (2015)	Singapore	GEO:GSE76427	Illumina GPL10558	Pair-matched HCC vs. adjacent liver tissues	115	8.656	0.064	52	8.009	0.065
Qin W et al. (2016)	China	GEO:GSE84402	Affymetrix GPL570	Pair-matched HCC vs. adjacent liver tissues	14	6.801	0.312	14	5.086	0.247
Hui KM et al. (2018)	Singapore	GEO:GSE121248	Affymetrix GPL570	Cirrhosis liver vs HCC	70	8.041	0.111	37	6.893	0.126
Wurmbach E et al. (2007)	USA	Oncomine:Wurmbach liver	Affymetrix GPL570	Cirrhosis liver vs. HCC and relapse tissues	35	8.049	0.234	40	6.263	0.088
Mas VR et al. (2009)	USA	Oncomine:Mas liver	Affymetrix GPL96	Cirrhosis liver vs. HCC and relapse tissues	64	6.463	0.109	60	6.080	0.084

Abbreviation: SD: standard deviation.

**Table 2:** Distribution of ATAD2 and clinicopathological characteristics from institutional HCC patients.

Characteristics	No. of patients	ATAD2		*χ* ^2^	*P*
		Low expression	High expression		
Tissue					
Tumor	80	28	52	8.046	0.006
Normal tumor	20	14	6		
Gender					
Male	66	25	41	1.374	0.357
Female	14	3	11		
Age (year)					
<50	25	7	18	0.783	0.454
≥50	55	21	34		
Tumor size					
≥5 cm	35	7	28	6.154	0.018
<5 cm	45	21	24		
Metastasis					
Yes	30	5	25	7.092	0.009
No	50	23	27		
Invasion					
Yes	27	6	21	2.925	0.136
No	53	22	31		
Cirrhosis					
Yes	76	27	49	0.185	1.000
No	4	1	3		
HBsAg					
Positive	64	22	42	0.055	1.000
Negative	16	6	10		
AFP					
<200 ng/dl	57	25	32	6.841	0.010
≥200 ng/dl	23	3	20		
TNM stage					
I + II	50	22	28	4.747	0.033
III + IV	30	6	24		
Differentiation					
WD	35	16	19	3.391	0.184
MD	35	10	25		
PD	10	2	8		

Abbreviations: WD: well differentiated; MD: moderately differentiated; PD: poorly differentiated.

**Table 3:** Distribution of ATAD2 and clinicopathological characteristics from TCGA LIHC patients.

Characteristics	No. of patients	ATAD2		*χ* ^2^	*P*
		Low expression	High expression		
Tissue					
Tumor	374	216	158	33.670	<0.001
Nontumor	50	50	0		
Gender					
Male	234	129	105	0.478	0.560
Female	110	65	45		
Age					
<50	70	32	38	4.077	0.058
≥50	274	162	112		
Race					
Asian	155	72	83	11.843	0.008
American Indian	2	1	1		
White	164	107	57		
Black	14	9	5		
Cirrhosis					
Negative	179	105	74	0.896	0.370
Positive	146	78	68		
Histological type					
HCC	334	185	149	4.904	0.086
ICC	7	6	1		
FL-HCC	3	3	0		
Family cancer history					
No	199	105	94	6.025	0.018
Yes	99	67	32		
Grade					
G1-G2	214	144	70	27.331	<0.001
G3-G4	130	50	80		
Tumor status					
Tumor free	222	124	98	0.004	1.000
With tumor	96	54	42		
Vascular tumor cell type					
None	194	118	76	2.650	0.109
Micro/macro	102	52	50		
Stage					
I-II	254	144	110	0.035	0.902
III-IV	90	50	40		
T stage					
T1-T2	258	147	111	0.142	0.709
T3-T4	86	47	39		
N stage					
N0	340	191	149	0.570	0.635
N1-N3	4	3	1		
M stage					
M0	339	190	149	1.150	0.392
M1	5	4	1		

Abbreviations: HCC: hepatocellular carcinoma; ICC: hepatocholangiocarcinoma; FL-HCC: fibrolamellar carcinoma.

## Data Availability

The data that support the findings of this study are openly available.

## Supplementary Materials

Supplementary Materials Table S1: univariate and multivariate analyses of individual parameters for correlations with overall survival rate: Cox proportional hazards model. Table S2: comparison of diagnostic efficacy among ATAD2, GPC3 and AFP in the diagnosis of HCC in Asian race from TCGA data. Figure S1: flow chart of the researching content in the present study. Figure S2: ATAD2 expression pattern based on Gene Expression Profiling Interactive Analysis (GEPIA) and genetic alteration from cBioPortal. Figure S3: ATAD2 protein in normal liver and HCC tissues from our institution. Figure S4: Kaplan-Meier survival curves from subgroup analysis of the relationships between ATAD2 expression and prognosis showing overall survival among TCGA (mRNA level) and CMUD (protein level) cases. A-H, CMUD; I-O, TCGA.
