# Supplementary material for: Integrated Bioinformatics Analysis of the Clinical Value and Biological Function of ATAD2 in Hepatocellular Carcinoma
Source: Biomed Res Int. 2020 May 5;2020:8657468. doi: 10.1155/2020/8657468 (PMC7225861; doi:10.1155/2020/8657468)
Supplement: Supplementary Materials — Table S1: univariate and multivariate analyses of individual parameters for correlations with overall survival rate: Cox proportional hazards model. Table S2: comparison of diagnostic efficacy among ATAD2, GPC3 and AFP in the diagnosis of HCC in Asian race from TCGA data. Figure S1: flow chart of the researching content in the present study. Figure S2: ATAD2 expression pattern based on Gene Expression Profiling Interactive Analysis (GEPIA) and genetic alteration from cBioPortal. Figure S3: ATAD2 protein in normal liver and HCC tissues from our institution. Figure S4: Kaplan-Meier survival curves from subgroup analysis of the relationships between ATAD2 expression and prognosis showing overall survival among TCGA (mRNA level) and CMUD (protein level) cases. A-H, CMUD; I-O, TCGA. [file 8657468.f1.zip › mat.8657468.v3.pdf]

Table S1: Univariate and multivariate analyses of individual parameters for correlations with overall survival rate: Cox proportional hazards model

Variables	HR	Univariate	*P*	HR	Multivariate	*P*
		CI (95%)			*CI* (95%)	
ATAD2	6.15	2.842-13.308	<0.001	2.49	1.007-6.161	0.048
Age	1.087	0.601-1.967	0.783			
Gender	1.627	0.833-3.177	0.154			
Size	1.763	1.018-3.054	0.043			
Metastasis	2.471	1.426-4.283	0.001	2.315	1.263-4.243	0.007
Invasion	1.349	0.773-2.354	0.292			
Cirrhosis	1.78	0.546-5.799	0.339			
HBsAg	1.367	0.697-2.683	0.363			
AFP	2.165	1.219-3.845	0.008	2.168	1.006-4.412	0.033
TNM	3.233	1.845-5.666	<0.001			
Differentiation	1.656	0.906-3.024	0.101			

Abbreviation:HR, Hazard Ratio;

Table S2:Comparison of diagnostic efficacy among ATAD2,GPC3 and AFP in the diagnosis of HCC in Asian race from TCGA data

Makers	Sensitivity(%)	Specificity(%)	Coincidence rate(%)	Area	*P*
ATAD2	83.02*	100	83.6(138/165)*	0.8878*	0.0013
GPC3	84.91*	100	85.5(141/165)*	0.9340*	0.0003
AFP	57.86	100	59.4(98/165)	0.6834	0.1224

Abbreviation:Compared to the AFP:$^{*}P<0.05$

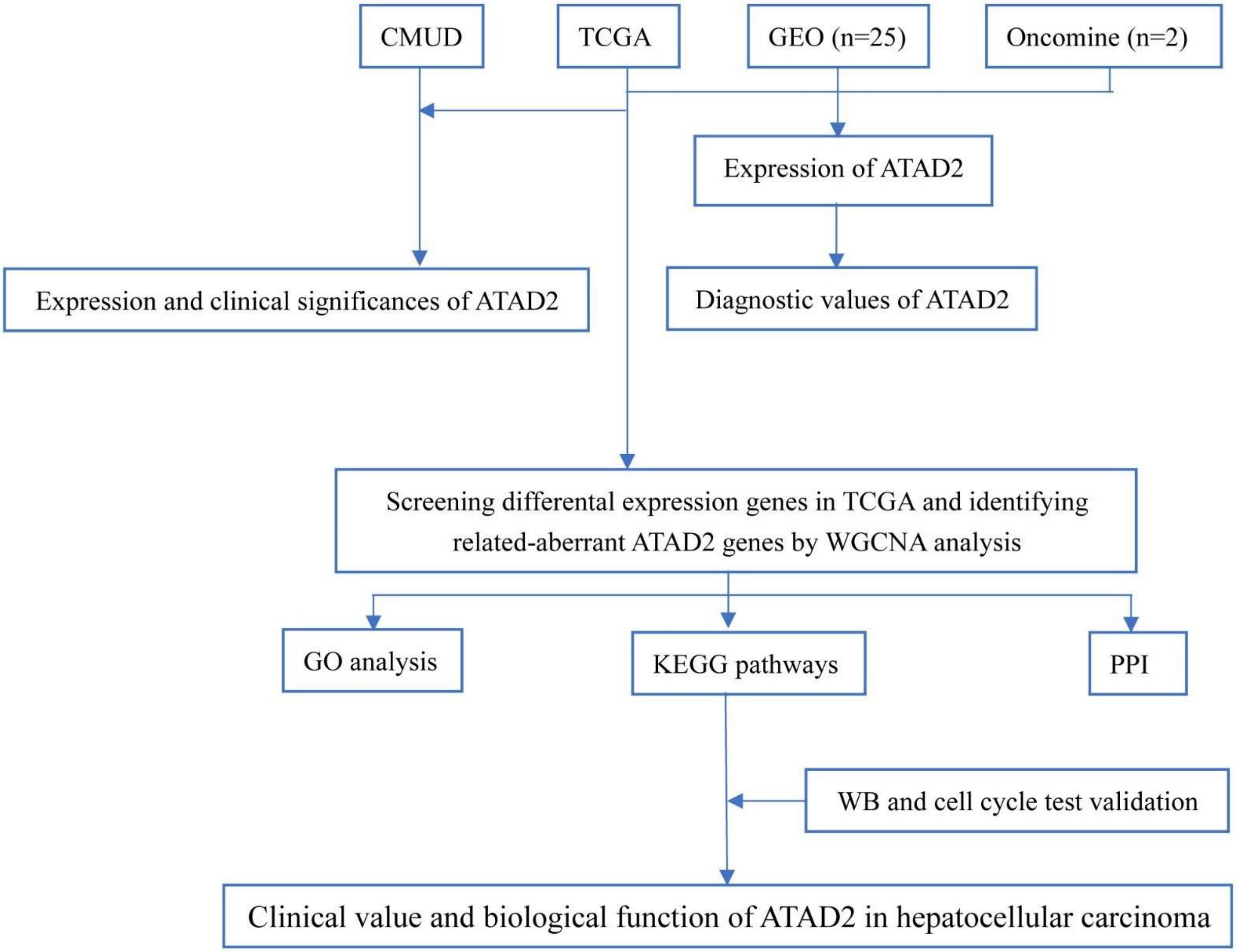

Figure S1: Flow chart of the researching content in the present study .

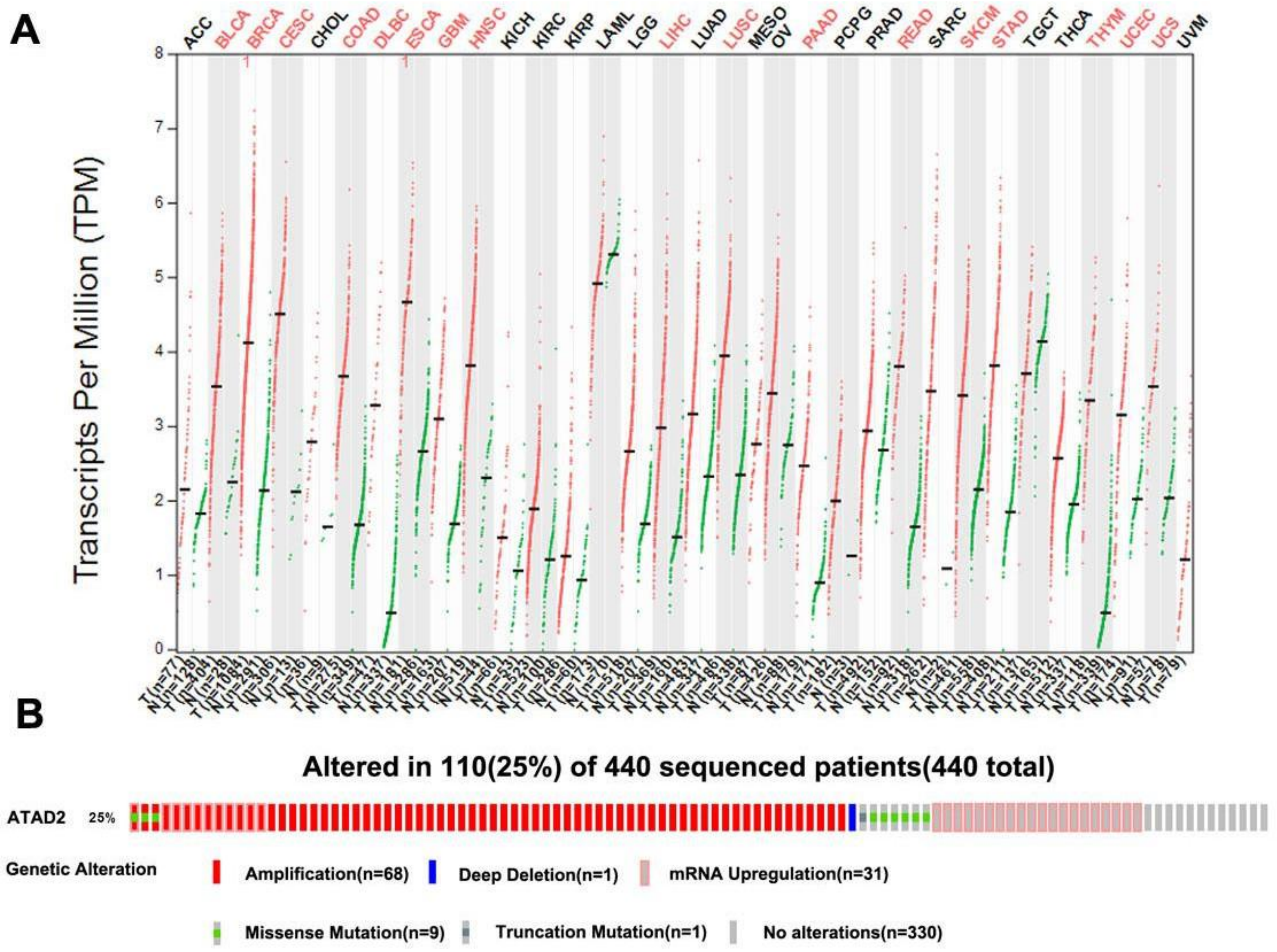

Figure S2 ATAD2 expression pattern based on Gene Expression Profiling Interactive Analysis (GEPIA) and genetic alteration from cBioPortal. A,Transcripts Per Million (TPM) data of ATAD2 expression are presented based on GEPIA.B, Genetic alteration of ATAD2 in 440 HCC patients from cBioPortal. ATAD2 was altered in a total of 110 HCC patients. ATAD2 amplificated in 68 patients and deep deleted in 1 patients. ATAD2 upregulated in 31 cases and its other alterings were 10 cases.

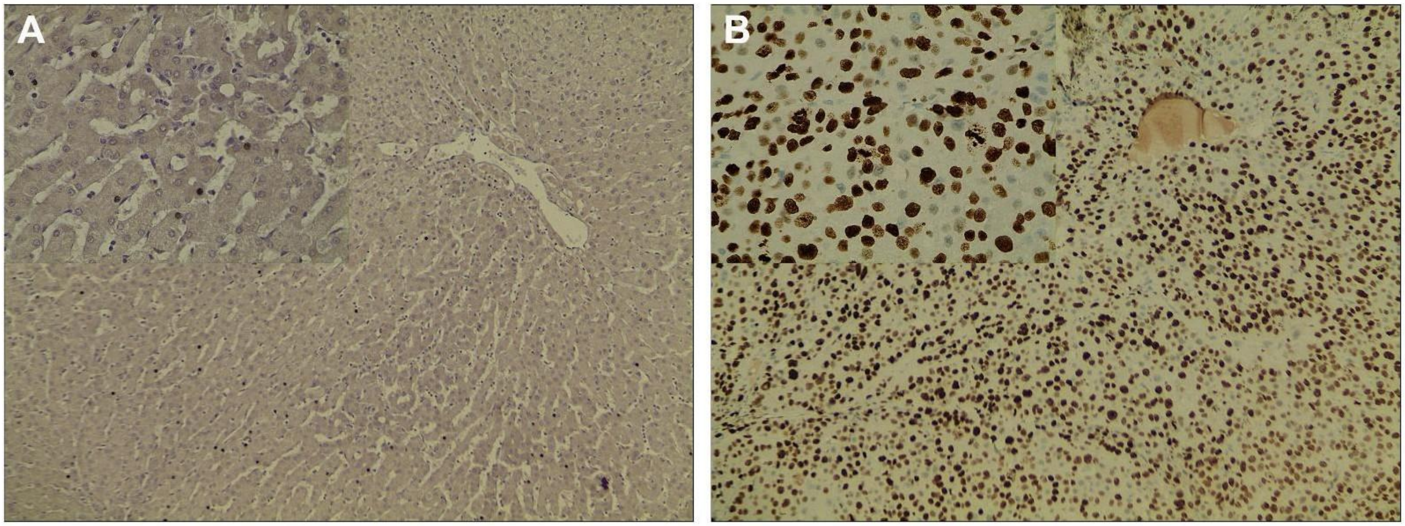

Figure S3 ATAD2 protein in normal liver and HCC tissues from our institution. A, Normal liver tissues stain negative for ATAD2, immunohistochemistry, ×200 (larger) ×400 (smaller); B, HCC tissues stain positive for ATAD2, immunohistochemistry, ×200 (larger) ×400 (smaller).

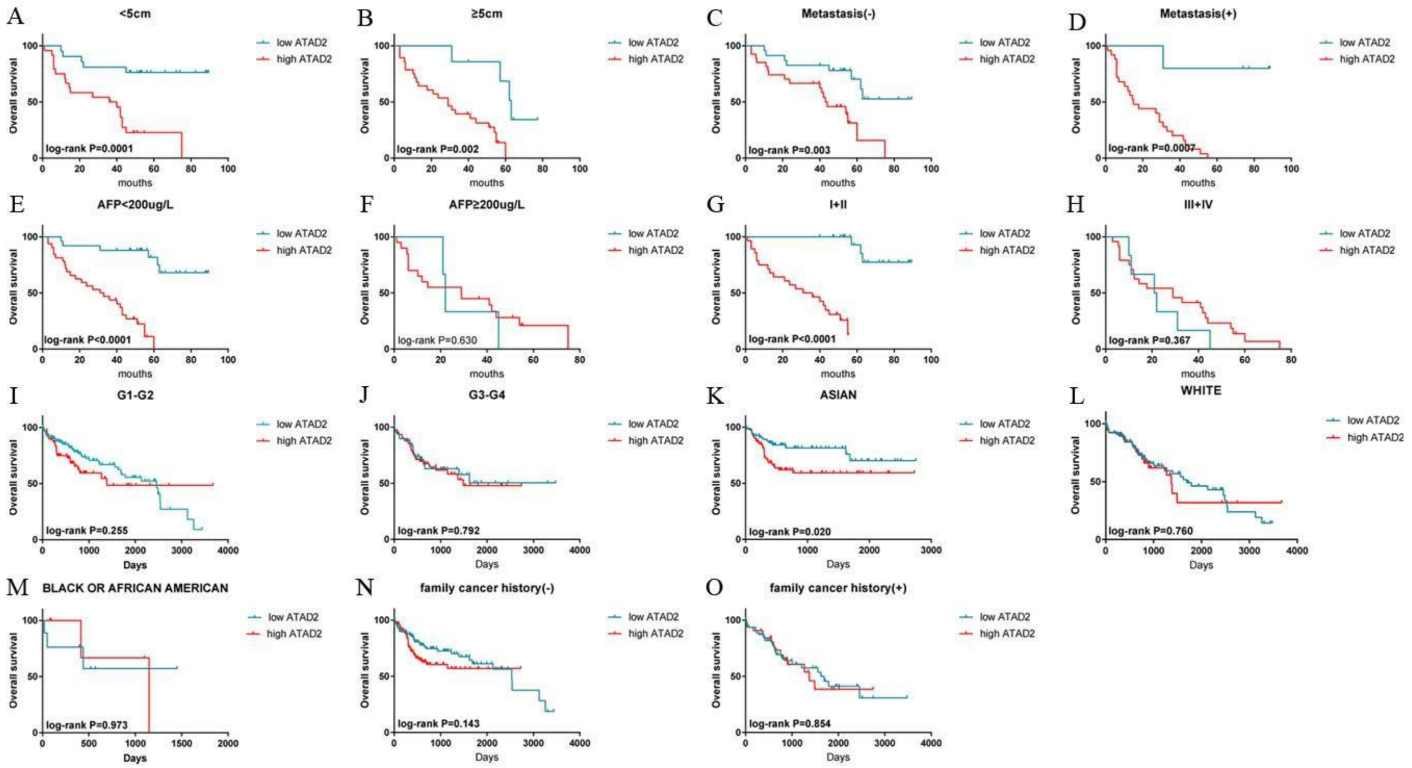

Figure S4 Kaplan-Meier survival curves from subgroup analysis of the relationships between ATAD2 expression and prognosis showing overall survival among TCGA (mRNA level) and CMUD (protein level) cases. A-H,CMUD; I-O,TCGA
